# Successful treatment of lamotrigine-induced Stevens–Johnson syndrome with upadacitinib

**DOI:** 10.1016/j.jdcr.2026.09.042

**Published:** 2026-09-30

**Authors:** Amishi Taneja, Jacob Kingsley, Severine Z. Cao, Jennifer B. Mancuso, Sara L. Fossum

**Affiliations:** a University of Michigan Medical School, Ann Arbor, Michigan; b Department of Dermatology, Michigan Medicine, University of Michigan, Ann Arbor, Michigan

**Keywords:** JAK inhibitor, lamotrigine, SJS, SJS/TEN, upadacitinib

## Introduction

Stevens–Johnsons Syndrome (SJS) and Toxic Epidermal Necrolysis (TEN) are on a spectrum of potentially fatal skin reactions, characterized by skin and mucosal loss due to keratinocyte death. Medications such as anticonvulsants, allopurinol, sulfonamides, and various antibiotics are major triggers.^1^

The immunopathogenesis involves complex mechanisms primarily mediated by cytotoxic CD8+ T cells, which ultimately induce keratinocyte apoptosis. More recently, studies identified the Janus kinase (JAK)-signal transduction and activation of transcription pathway as a key pathogenic driver in SJS/TEN.^2^, ^3^ In vitro JAK inhibitor usage showed decreased cytotoxicity towards keratinocytes, highlighting their utility as a potential novel treatment option in SJS/TEN. Seven patients with TEN treated with off-label JAK inhibitors together with systemic corticosteroids all survived at 30 days, and signs of reepithelialization were demonstrated just 4 days after administration.^3^ In subsequent case reports, JAK inhibitors have shown efficacy when paired with systemic corticosteroids, but the efficacy of monotherapy remains unclear.^4^, ^5^ There is limited data on the efficacy and safety of JAK inhibitors compared to different treatments in SJS/TEN.^6^

We present a case of lamotrigine-induced early SJS treated with upadacitinib, which contributes to the paucity of literature on JAK inhibitors as an emerging targeted therapy for SJS/TEN.

## Case report

A 33-year-old woman presented with a new, progressive rash approximately 2 weeks after starting lamotrigine for refractory depression. Her rash first appeared on the trunk with subsequent spread to the face and extremities over 24 hours. Symptoms included pruritus, which evolved into burning and tenderness. She also reported oral pain, dysuria, and vulvovaginal pruritus. Ocular symptoms included increased tearing and difficulty opening the eyes first thing in the morning. One week before rash onset, she experienced a flu-like prodrome of nausea, vomiting, and sore throat.

On the evening of rash onset, she presented to an outside emergency department where she received intravenous methylprednisolone (dosage not documented) and diphenhydramine. She was discharged home; however, her rash and symptoms continued to worsen over the next day, at which time she presented to our institution and dermatology was consulted.

On exam, diffuse erythematous macules and papules coalescing into larger patches and plaques were seen involving the chest, abdomen, back, buttocks, arms, and legs (Fig 1, *A*-*D*). Excoriations were present without sloughing or blistering. Nikolsky sign was negative. Oropharyngeal exam revealed a single erosion on the soft palate. Conjunctival injection was present.

**Fig 1:**
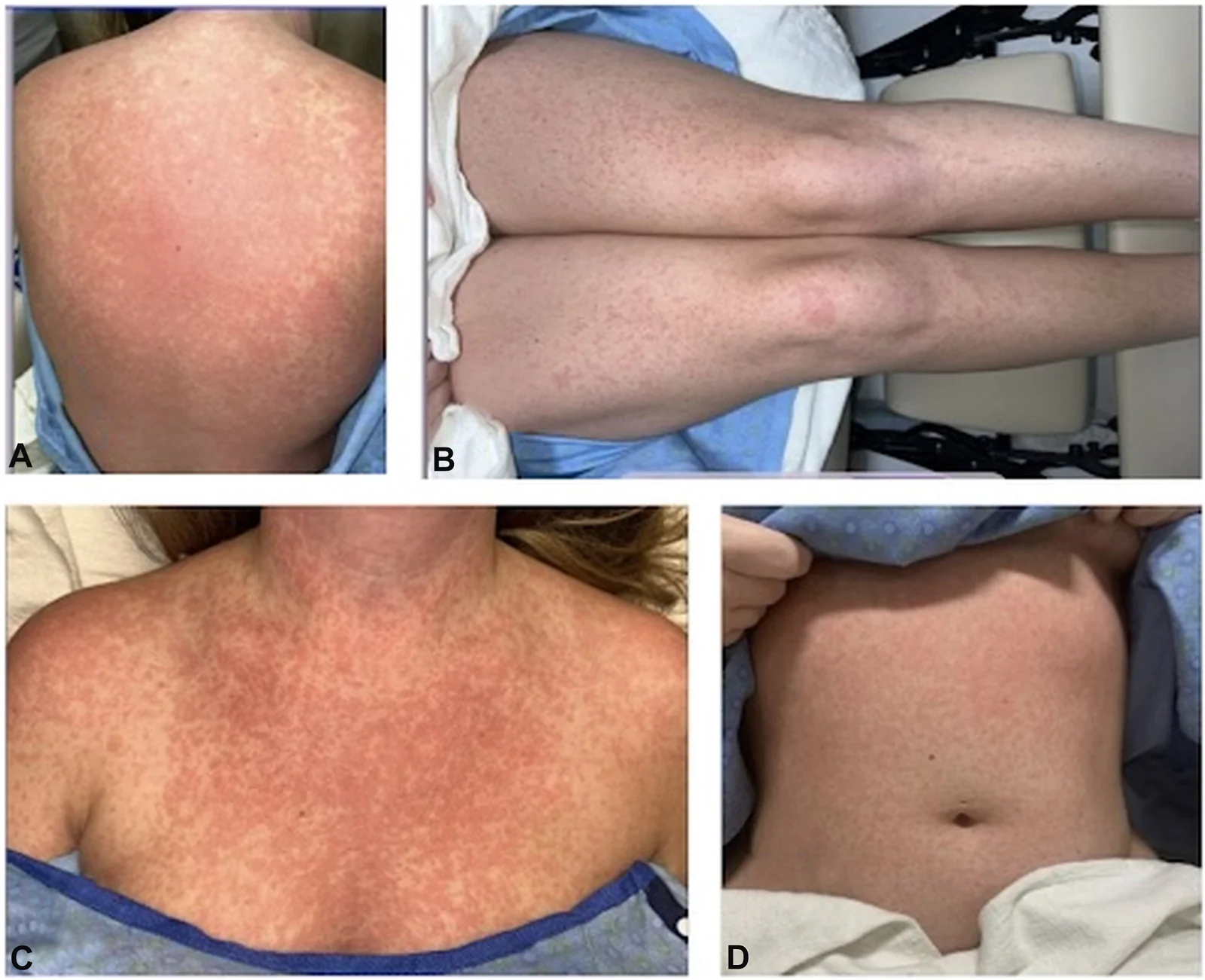
Exam at presentation revealed diffusely scattered erythematous macules and papules coalescing into larger patches and plaques involving the back **(A)**, arms, legs **(B)**, chest **(C)**, abdomen **(D)**, and buttocks.

The patient was afebrile and otherwise hemodynamically stable. Laboratory evaluation was notable for mild leukocytosis without peripheral eosinophilia. Comprehensive metabolic panel demonstrated normal renal and hepatic function. On Day 1 of presentation, her Severity-of-Illness Score for Toxic Epidermal Necrolysis (SCORTEN) was 0. A 4-mm punch biopsy was obtained from the back for routine histopathology, which showed a superficial perivascular dermatitis with sparse neutrophils and foci of basal vacuolization, consistent with early SJS. Additional laboratory studies, including hepatitis serologies, human immunodeficiency virus screening, and tuberculosis testing, were within normal limits.

Given the patient’s recent initiation of lamotrigine, the diffuse morbilliform eruption accompanied by pruritus and pain, and early signs of mucosal involvement, her clinical picture was highly concerning for early SJS. This was further supported by her flu-like prodrome preceding the rash, which is common in SJS/TEN.^7^

On day 1 of hospitalization, lamotrigine was discontinued. Ophthalmology was consulted, and slit lamp examination revealed conjunctival injection with no corneal or conjunctival staining. Gynecology was also consulted, and pelvic examination was significant for erythematous vaginal mucosa within the introitus.

Given the high clinical suspicion for SJS, the absence of comorbidities, and the early stage of her disease, the decision was made to initiate upadacitinib 30 mg daily without additional systemic corticosteroids. She received symptomatic treatment with topical triamcinolone and clobetasol twice daily, plus hydroxyzine as needed for pruritus.

In the first 2 days of treatment, her rash initially worsened on the legs but improved elsewhere. Her SCORTEN remained at 0 on day 3. By day 6, she was entirely clear of rash (Fig 2, *A*-*C*). There were no ocular or vulvovaginal complications. On day 7, she was discharged after receiving 7 total days of upadacitinib therapy.

**Fig 2:**
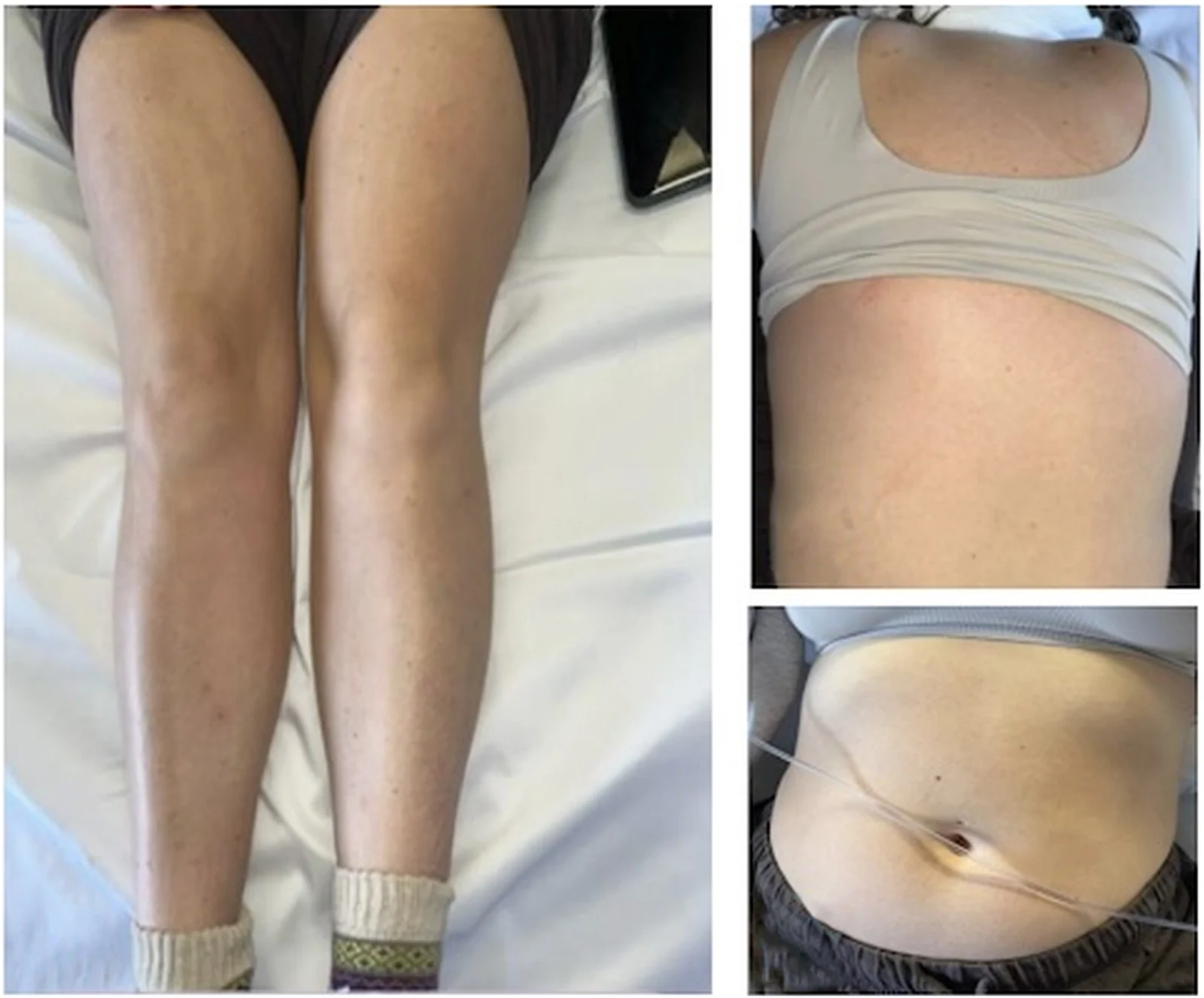
The patient was entirely clear of rash at discharge.

## Discussion

This case highlights the successful treatment of lamotrigine-induced early SJS with upadacitinib for 7 days. Notably, our patient did not receive coadministrated systemic corticosteroids as part of her treatment regimen at our institution, though she did receive a single dose of intravenous methylprednisolone at an outside emergency department. This may have contributed to her clinical improvement; however, the rapid resolution of her mucocutaneous disease without the need for additional high dose intravenous steroids does suggest a role for upadacitinib in treatment of SJS.

The use of JAK inhibitors in managing SJS/TEN is supported by recent studies demonstrating the importance of JAK/signal transduction and activation of transcription pathways as key drivers of keratinocyte death. Which JAK inhibitor is most efficacious remains to be seen. In mouse models, oral doses of pan-JAK inhibitors (tofacitinib), JAK1/JAK2 inhibitors (baricitinib), and JAK1-specific inhibitors (abrocitinib and upadacitinib) showed marked improvement in clinical and histological disease.^3^ Two case reports showed successful treatment of SJS/TEN with upadacitinib and methylprednisolone, with complete reepithelialization in both patients.^4^, ^5^

A Cochrane systematic review found limited high-quality evidence that systemic immunomodulatory medications decrease mortality rates in SJS/TEN.^8^ There is a great need to identify evidence-based treatments to improve morbidity and mortality in patients with SJS/TEN, and to further explore the use of JAK inhibitors in treatment.

Our patient presented to us within days of rash onset and had an early case of SJS with a low baseline SCORTEN. Early administration of the JAK inhibitor, at 2 days after rash onset, likely prevented her from developing epithelial detachment and more severe mucositis. While her low SCORTEN already corresponded to a favorable prognosis, the rapid response within days of initiating upadacitinib highlights the utility of early JAK inhibitor use in SJS/TEN. Future studies will need to explore the use of JAK monotherapy in patients.

Although further studies are needed, this case adds to emerging data supporting the use of JAK inhibitors as targeted therapy for SJS/TEN and suggests early initiation before epidermal loss may be most beneficial in preventing epidermal necrosis and detachment.

### Declaration of generative AI and AI-assisted technologies in the writing process

None.

## Conflicts of interest

None disclosed.
